# A Method for Reconstructing Background from RGB-D SLAM in Indoor Dynamic Environments

**DOI:** 10.3390/s23073529

**Published:** 2023-03-28

**Authors:** Quan Lu, Ying Pan, Likun Hu, Jiasheng He

**Affiliations:** School of Electrical Engineering, Guangxi University, Nanning 530004, China

**Keywords:** indoor dynamic environments, visual SLAM, camera pose, randomized ferns, keyframes, 3D reconstructing

## Abstract

Dynamic environments are challenging for visual Simultaneous Localization and Mapping, as dynamic elements can disrupt the camera pose estimation and thus reduce the reconstructed map accuracy. To solve this problem, this study proposes an approach for eliminating dynamic elements and reconstructing static background in indoor dynamic environments. To check out dynamic elements, the geometric residual is exploited, and the static background is obtained after removing the dynamic elements and repairing images. The camera pose is estimated based on the static background. Keyframes are then selected using randomized ferns, and loop closure detection and relocalization are performed according to the keyframes set. Finally, the 3D scene is reconstructed. The proposed method is tested on the TUM and BONN datasets, and the map reconstruction accuracy is experimentally demonstrated.

## 1. Introduction

Camera-based 3D reconstruction acquires the image data of objects using vision sensors and then reconstructs information, such as textures and surface contours, of the objects in real-world environments using relevant theories. Three-dimensional reconstruction technology plays an important role in scenarios, such as artificial intelligence, robot navigation, autonomous driving, virtual reality, and 3D printing. With the spread of commercial RGB-D cameras and the development of the graphics processing unit, 3D dense reconstruction has become widely studied in the field of visual Simultaneous Localization and Mapping (SLAM). Several related studies have finally led to satisfactory results [1,2,3,4,5,6,7].

With the advent of commercial RGB-D cameras, the dense reconstruction of 3D scenes using RGB-D images has been widely studied in visual SLAM. The RGB-D camera provides both a color image and a depth image. The depth image provides the distance of each pixel from the camera. Using the distance of the pixel points and their position in the image coordinates, the 3D spatial coordinates of each pixel point can be calculated and a 3D scene can be reconstructed. Since its release in 2010, the Kinect camera has attracted a lot of attention. It has been used in research for 3D reconstruction. The KinectFusion [8] camera-based 3D reconstruction integrates depth data from the Kinect camera into a Truncated Signed Distance Function (TSDF) model, and uses the Iterative Closest Point (ICP) to obtain camera pose in real time. It performs a basic model reconstruction. However, its reconstruction is limited to small scenes. The ElasticFusion [9] system combines local loop closure detection and global loop closure detection. It ensures the global consistency of the reconstruction results to some extent. However, it is also applicable to small-scale scenarios. The BundleFusion algorithm [10] presents a parallelized framework that uses the sparse feature, dense geometry, and luminosity matching correspondence to estimate the bundle adjustment in real time with a relocation ability.

This reconstruction process has been performed by assuming that the robot’s working environment is static, while the real circumstances often contain dynamic factors. To the best of our knowledge, there is no new visual SLAM solution specifically proposed to address the interference of dynamic factors in dynamic environments. The existing reconstruction method for dynamic environments is based on the existing static framework, where the front end visual odometry removes the dynamic factors and then uses the static points in the environment to calculate the poses between neighboring cameras and construct an environment map. Most of the visual SLAM solutions [11,12,13,14,15,16,17] for dynamic environments mainly focus on the localization, while relatively few studies focus on the reconstruction. The ReFusion [18] is based on the TSDF model, which uses geometric residuals to distinguish between dynamic and static factors, and then rejects the dynamic factors. Although this study tackles the 3D reconstruction of static scenes, it still focuses on the camera pose estimation without optimization of the reconstructed map. The StaticFusion [19] uses the static pixel probability of the current frame to distinguish between dynamic and static factors, with the disadvantage that the initial static surface map cannot contain a large number of dynamic objects to ensure a high accuracy. The PoseFusion [20] uses human joints as a priori knowledge for human life scenarios, performs a minimum cut in point cloud data to obtain human regions and then reject them, and finally, it develops a dynamic dense slam system based on ElasticFusion [9]. The Flowfusion [21] performs dense optical flow computation based on the PWC Net [22], where the obtained scene flow region is the dynamic object, and the static background reconstruction is performed by iterations after removing the dynamic factors. With the development of deep learning, many studies use it to add semantic information to SLAM systems in dynamic environments based on networks such as SegNet [23], Mask R-CNN [24], and YOLO [25], and the use of the a priori knowledge can initially judge and segment the moving objects in the environment. Although these methods accurately perform 3D reconstruction, they do not optimize the reconstructed maps.

The traditional 3D reconstruction technology for indoor environments assumes that the robot is in an ideal environment where the object is in a static, rigid body without clear light changes or human interference with the scene. However, there are various dynamic factors in the actual environment, such as moving persons or objects. In the static environment, the objective function can be developed based on the geometric constraints between the camera motion trajectory and the static pixel points to find the camera pose and construct the static map. In dynamic environments, the traditional visual SLAM solutions cannot distinguish whether the robot itself is moving or the objects present in the environment are moving. In addition, the environmental obscuration caused by the motion of dynamic factors can make the feature matching wrong, which significantly affects the camera pose estimation and loop closure detection and greatly reduces the feasibility of the algorithm or even causes it to fail.

To solve this problem, this study proposes a method for filtering out the dynamic factors and reconstructing the static background map. The proposed method is tested on the TUM and BONN datasets, and the map reconstruction accuracy is experimentally demonstrated.

The contributions of this paper are summarized as follows:

(1) A front-end visual odometer that uses geometric residuals to remove dynamic factors and embeds the processed images into the BundleFusion framework, which allows the static environment-based system to handle dynamic environments.
(2) The introduction of randomized ferns to select keyframes effectively decreases the negative impact of residual dynamics on the loop closure detection and relocalization.
(3) The proposed study improves the map accuracy.

The remainder of this paper is organized as follows: Section 1 introduces the challenges of 3D reconstruction in dynamic environments and related works. The materials and methods are detailed in Section 2. Section 3 presents the results of experiments. Finally, the discussion and conclusions are drawn in Section 4 and Section 5, respectively.

## 2. Materials and Methods

This section describes the proposed methodology. It also describes the hardware and software platforms and datasets required for the experiments.

### 2.1. Algorithm Overview

**Figure 1:**
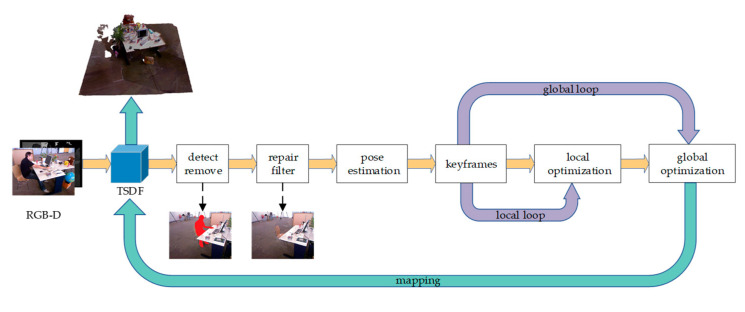
Overview of the proposed RGB-D SLAM system based on BundleFusion.

Figure 1 shows an overview of the proposed RGB-D SLAM system based on BundleFusion. Based on the registration of the TSDF [26] model, the geometric residuals are first used to detect and eliminate the dynamics in the image. The region-growing approach is then used to restore the image. The restored images are input into the front end of the BundleFusion [10] as a pre-processing stage. Afterward, the obtained static background is subjected to camera pose estimation. In contrast to BundleFusion, the randomized fern is introduced in the selection of keyframes to reduce the impact of the incompletely filtered dynamic fragments on the 3D reconstruction. The local optimization and global optimization modules, respectively, perform local loop closure detection and global closure detection, which reduces the bias of camera estimation with time and space variations.

### 2.2. Model Representation

The Truncated Signed Distance Function is used to rebuild a 3D dense map. Voxel grid is the core of the TSDF. The algorithm consists of dividing the whole 3D space to be reconstructed into grids, and each grid stores the values such that the negative and positive distances to the nearest surface point, respectively, correspond to the inner and outer voxels of the surface, and the surface itself is defined as the over-zero point in SDF. When the distance of a voxel from the surface is greater than a certain threshold, its SDF value is ignored (i.e., the sign distance is truncated). Each voxel is projected into the image plane, its depth relative to the camera is compared with the nearest pixel in the depth image, and the result of this comparison is denoted by $D_{n}(x)$. In addition, to improve the robustness of the system, each voxel stores the weight value w, as well as the color information $C_{n}(x)$. Those voxel values are then updated as follows:

(1) $$D_{n}{\left(x\right)}_{n+1}=\frac{D_{n}(x)W_{n}(x)+{\overset{\wedge }{D}}_{n}(x)\overset{\wedge }{W}(x)}{W{\left(x\right)}_{n}+\overset{\wedge }{W}(x)}$$

(2) $$C{\left(x\right)}_{n+1}=\frac{C{\left(x\right)}_{n}W{\left(x\right)}_{n}+C(x)W(x)}{W{\left(x\right)}_{n}+W(x)}$$

(3) $$W{\left(x\right)}_{n+1}=\min(W{\left(x\right)}_{n}+\overset{\wedge }{W}(x),W_{\max})$$

where ${\overset{\wedge }{D}}_{n}(x)$ is the estimate of x.

### 2.3. Pose Estimation

Each RGB-D image frame consists of color information and depth information. Assuming that the pixel coordinate of a pixel p is represented by $p={\left[\begin{array}{cc} u & v \end{array}\right]}^{T}$, the depth function is defined as $Z(p):{\mathbb{R}}^{2}\to \mathbb{R}$, and the intensity function, which is relative to the color, is denoted by $I(p):{\mathbb{R}}^{2}\to \mathbb{R}$. The mapping relationship between pixel points and 3D points in space can then be expressed as follows:

(4) $$x=\left[\begin{array}{c} \frac{u-c_{x}}{f_{x}}Z(p)\\ \frac{v-c_{y}}{f_{y}}Z(p)\\ Z(p) \end{array}\right]$$

The intrinsic parameters of the camera are $c_{x}$, $c_{y}$, $f_{x}$, and $f_{y}$. The transformation $T\in {\mathbb{R}}^{4\times 4}\in SE(3)$ is given by the following equation:

(5) $$T=\left[\begin{array}{cc} R & t\\ 0^{T} & 1 \end{array}\right]$$

where $R\in {\mathbb{R}}^{3\times 3}\in SO(3)$ is the rotation of the camera, and t denotes the translation.

A small rigid body motion can be represented by $\xi =(w_{1},w_{2},w_{3},v_{1},v_{2},v_{3})$, where $\left(w_{1},w_{2},w_{3}\right)$ is the rotation of the camera and $\left(v_{1},v_{2},v_{3}\right)$ denotes the translation. In the TSDF model, the depth-dependent error function is expressed as the following:

(6) $$E_{d}=\sum_{i=1}^{N}{\left|\right|D(\exp(\overset{\wedge }{\xi })Tx_{i})\left|\right|}^{2}$$

The error function associated with the color is given by the following:

(7) $$E_{c}(\overset{\wedge }{\xi })=\sum_{i=1}^{N}{\left|\right|C(\exp(\overset{\wedge }{\xi })Tx_{i})-I(p_{i})\left|\right|}^{2}$$

The joint error function is expressed as the following:

(8) $$E(\overset{\wedge }{\xi })=E_{d}(\overset{\wedge }{\xi })+w_{c}E_{c}(\overset{\wedge }{\xi })$$

Therefore, solving for the camera pose can be converted into finding the minimum error function as follows:

(9) $$\xi^{*}=\arg\min E(\xi )$$

### 2.4. Loop Closure Detection and Relocalization

The dynamics are not always completely filtered out, and thus the background restoration of images containing dynamics is not always satisfactory. Therefore, loop closure detection and relocalization are crucial in dynamic visual SLAM. For example, PoseFusion [20] only focuses on detecting dynamic objects with the human body as the target and cannot judge the moving objects in the environment. In addition, deep learning trains a limited number of a priori dynamic object types, which can easily lead to failure of camera tracking when untrained dynamic objects are present in the environment, which ultimately causes a reduction in reconstruction accuracy or even leads to reconstruction failure.

The current mainstream studies on the improvement of the visual SLAM robustness for dynamic environments focus on optimizing the camera pose. However, it does not take into consideration the background reconstruction. Some studies [18,19] only embed the front-end vision with the dynamic factors that are directly removed into the existing SLAM 3D reconstruction framework without optimizing their fusion. This study focuses on reducing the adverse effects of residual dynamic factors on the 3D reconstruction from loop closure detection and relocalization.

Similar to BundleFusion, this study treats every 10 frames as a submodule. The difference is that BundleFusion performs 3D reconstruction based on a static environment, which treats the first frame of each submodule as a keyframe for loop closure detection and relocalization. However, it cannot be ensured that the first frame of each submodule after dynamic factor filtering and background restoration is completely free of dynamic factor residues, and therefore the first frame is not necessarily a suitable keyframe. Therefore, in this study, the frame in the first module without dynamic factor residue is considered the first keyframe, and if there is no eligible keyframe in the first module, it is discarded, and the process continues down until the first keyframe is found.

The filtered dynamic factors are fed into the BundleFusion framework. When matching feature points, a matching error occurs if the dynamic factors in the image are not fully filtered. Similar to the DMS-SLAM [27], the matching error caused by dynamic objects is analyzed. The difference is that in this work only the potential keyframes are analyzed. A grid n×n constructed with each feature point as the center is considered a correct match if it matches the feature point in the reference frame in the same region of the current image frame, and vice versa. If the number of errors exceeds a set threshold t, the image is considered to have a high residual dynamic factor and is not suitable as a keyframe.

**Figure 2:**
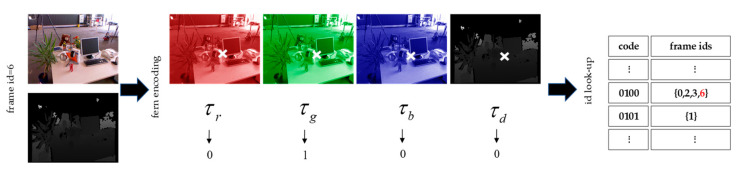
Random fern coding schematic.

Subsequent keyframes are determined based on the randomized ferns. To select keyframes, the image features of a frame of RGB-D images can be coded using the randomized ferns. As shown in Figure 2, a randomly selected position in an input RGB-D image encoding the entire frame in binary, while each fern generates a small block of encoding, and each block points to a row of the encoding table. New images are continuously acquired. If the dissimilarity is greater than a certain threshold, the id of the new incoming frames will be added to the row.

After finding the first keyframe, the keyframes of the subsequent submodules are determined using randomized ferns and added to the keyframe set. In the determination process of the subsequent keyframes, the RGB-D image is first encoded using a random fern as its feature information. The similarity between the current frame and the keyframe is then calculated based on the defined BlockHD as the similarity measure, and whether to add this frame to the keyframes set is determined. When loop closure detection is performed, if the BlockHD value of the current frame and the key frame is less than a predefined threshold δ, it is considered that a loop closure is detected, and the camera pose is corrected accordingly. In addition, when the camera pose estimation fails, the camera is relocated by retrieving the camera pose corresponding to similar keyframes:

(10) $$BlockHD(b_{C}^{I},b_{C}^{J})=\frac{1}{m}\sum_{k=1}^{m}b_{F_{k}}^{I}\equiv b_{F_{k}}^{J}$$

where $b_{C}^{I}$ and $b_{F_{k}}^{I}$ are, respectively, the encoding and binary encoding blocks of frame I, $b_{C}^{J}$ and $b_{F_{k}}^{J}$ are the encoding and binary encoding blocks of frame J, respectively. Note that the smaller the BlockHD, the more similar the images, and the greater the discrepancy.

### 2.5. Local Optimization and Global Optimization

As with BundleFusion, a hierarchical optimization strategy is used. It consists of applying local optimization within each submodule and global optimization between the submodules. The difference is that scale-invariant feature transform (SIFT) descriptors are not used for feature matching. Because the 3D reconstruction based on dynamic environment will inevitably leave some dynamic factors, if the SIFT feature points happen to be the dynamic factors, they will lead to too much SIFT feature offset, which makes the reconstructed 3D structure have large error. The pose graph optimization is used to perform local optimization for local loop closure detection, and global optimization for global loop closure detection.

### 2.6. Platform and Dataset

The experiments were run on a desktop computer equipped with an Intel Core i5 CPU, 16 GB of RAM, and an NVIDIA GeForce RTX3070 graphics card. The Technical University of Munich (TUM) dataset [22] and the BONN dataset are indoor dynamic environment sequences captured using Microsoft Kinect. The difference is that the BONN dataset provides a ground-truth 3D model, while each scene targets a single dynamic feature. The TUM dataset does not include large, realistic ground models, but the scenes contain more distinct dynamic features. The freiburg2_desk_with_person_validation sequence in TUM dataset was used to demonstrate the details of the experimental method. In addition, the BONN dataset of ballon, ballon_tracking, crowd, kidnapping_box, mov-ing_nonobstructing_box, moving_obstructing_box, person_tracking, plac-ing_nonobstructing_box, placing_obstructing_box, removing_nonobstructing_box, re-moving_obstructing_box, and synchronous sequences were used to evaluate the accuracy of the reconstructed map.

## 3. Results

In this section, the experiments are designed to validate the method proposed in Section 2. The performance of the proposed method, both qualitatively and quantitatively, is demonstrated by the experimental results.

### 3.1. Qualitative Results

**Figure 3:**
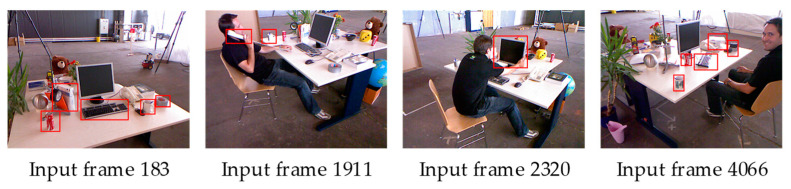
RGB frames from the TUM dataset containing dynamics.

The experimental effect of the freiburg2_desk_with_person_validation sequence in the TUM dataset was first validated. In this sequence, a person walks close to the desk and sits down, and moves the objects on the desk from time to time, as shown in Figure 3. Some objects on the desk are moved to different positions over time. The objects that are being moved in the red boxes and the person who is moving the objects are dynamic objects in the sequence.

**Figure 4:**
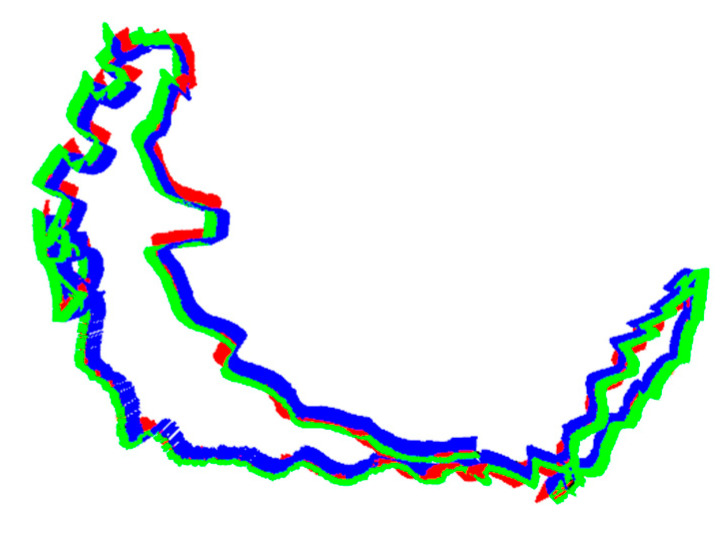
Camera trajectory.

The sequence provides a decent test of the robustness of the SLAM system to dynamic factors. In addition, the camera motion mode makes the sequence have a complete loop closure, which can assess the loop closure detection and relocalization function. Figure 4 shows the camera trajectory. Red, green, and blue lines are the x, y, and z axes of each camera pose respectively. The trajectory diagram shows that the camera makes a complete circle and forms a closed loop.

**Figure 5:**
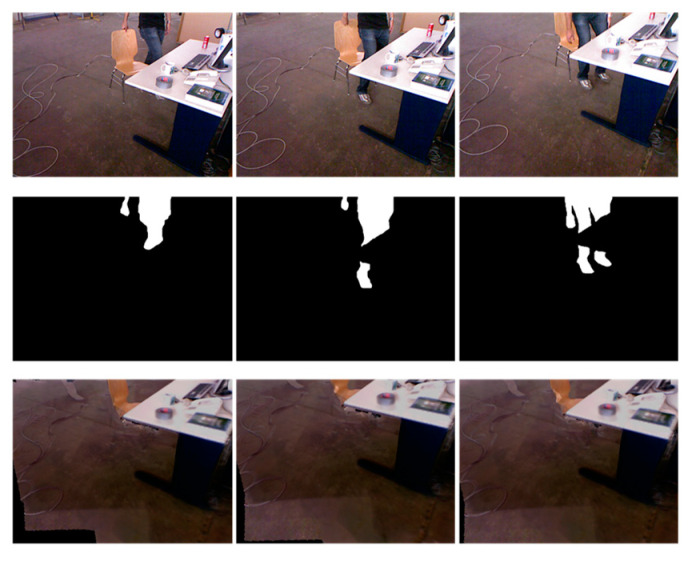
Background inpainting results.

The dynamic factors are first detected frame by frame for the input sequence using the geometric residual method. The core idea of the TSDF model is to represent the world with a 3D voxel grid in which each voxel contains an SDF value. The SDF is a function $V_{SDF}(x):{\mathbb{R}}^{3}\to \mathbb{R}$ that returns, given a point in space, its distance to the nearest surface. Similar to ReFusion, the residual of the *i*-th pixel in the image is defined as $r_{i}=V_{SDF_{i}}^{2}$. Given a threshold τ, if $r_{i}>\tau$, the pixel is part of a dynamic object. They are then filtered and background-restored using a region-growing method similar to that in [28], as shown in Figure 5. The detection of dynamic factors using the geometric residual method may suffer from incomplete detection of the same dynamic object. The basic idea of the region-growing method is to merge pixel points with similar properties; therefore, the introduction of region growing enables the complete detection of dynamic objects and their segmentation. Region growing is performed based on point attribute similarity within the detected dynamic object region, where the point data lack a clear neighborhood relation. Frames 1125, 1136, and 1148 were selected to display the dynamic changes. Figure 5 shows a person walking to the table and preparing to sit down. The first row shows the original frames. The original image was aligned to the TSDF model using the method described in Section 2.1, and the dynamic factors from the image were detected and filtered out using the geometric residual method. The second row shows the mask of the dynamic factors. Finally, the images is are restored and the dynamic factors are removed. The third row shows the images after restoration of the background.

**Figure 6:**
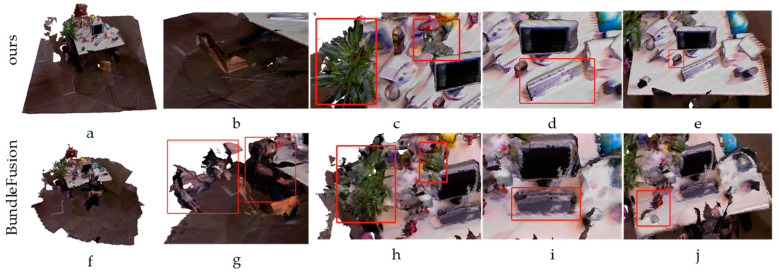
Top: the reconstruction result obtained by the proposed method. Bottom: the reconstruction result obtained after filtering out the dynamics and inputting it directly into BundleFusion. (**a**,**f**) are the reconstruction effects of the proposed method and BundleFusion, respectively. (**g**) is a sequence scene in which the moving people are not cleanly filtered out, and the corresponding figure (**b**) can filter out the moving people. (**c**) is a clearer reconstruction of the potted plants in the sequence than figure (**h**). (**d**) is the position of the keyboard after it is moved by a human, while (**i**) is the position before the keyboard is moved. The position of the keyboard in (**e**) is the position of coke after it is moved, while the position tracking failure of (**j**) for coke leads to the reconstruction of a coke bottle with continuous fragments, similar to the tracking effect of figure g for people.

The loop closure detection and relocalization module is based on BundleFusion, but it differs in the selection of keyframes that are less likely to be disturbed in static scenes. However, it is based on a dynamic environment, and therefore, there will inevitably be some dynamic factors that cannot be completely filtered out. If the key point is the dynamic factors, it will greatly reduce the algorithm’s performance. The filtered dynamic factor image is input directly into BundleFusion for experimental comparison with the improved loop closure detection and relocalization model, as shown in Figure 6. 

The proposed method can be used for 3D reconstruction in dynamic scenes after filtering out dynamic factors and improving the relocation module based on the BundleFusion framework.

**Figure 7:**
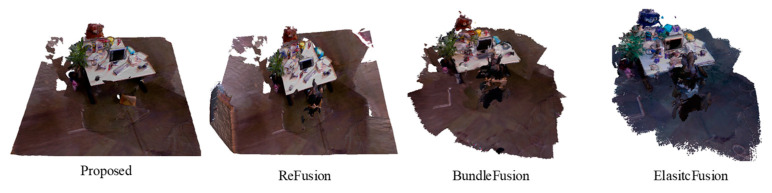
Reconstruction results obtained by four different methods.

The existing SLAM solutions for dynamic environments mostly focus on reducing the effect of the dynamics on camera pose estimation, while few studies on 3D scene reconstruction after filtering out dynamics exist. The ReFusion focuses on camera pose estimation, and it also provides 3D reconstruction results. The reconstruction results obtained by the proposed method are compared with those obtained by ReFusion and those after filtering out dynamic factors as the input sources of BundleFusion and ElasticFusion. The obtained results are shown in Figure 7. It can be seen that ReFusion, BundleFusion, and ElasticFusion do not process the incompletely filtered dynamic factors, causing the reconstruction results to have many incompletely filtered dynamic fragments.

### 3.2. Quantitative Results

**Figure 8:**
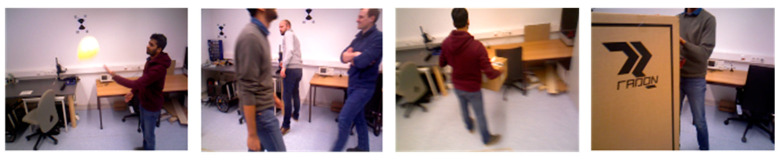
Example RGB frames from Bonn dataset.

The trajectory error is used in most of the studies to evaluate the map reconstruction accuracy. However, the proposed method is map-centric. The evaluation in this manuscript therefore focuses on the accuracy of the 3D map reconstruction. In this study, the evaluation metric proposed by Handa et al. [29] was used. For the experiment, 10 dynamic sequences were selected from the Bonn RGB-D Dynamic Dataset proposed by the Photogrammetry and Robotics Lab of the University of Bonn. These dynamic sequences contain different complex scenarios such as one person moving, several people moving, one person moving a box, and two people moving a box together. Figure 8 shows an example of some of the sequences.

This dataset provides a ground-truth model that enables an evaluation of the reconstruction accuracy using the above-mentioned method. Several available open-source algorithmic frameworks, including ReFusion, BundleFusion, and ElasticFusion, were used for comparison. The obtained reconstruction results are presented in Table 1.

ReFusion provides reconstructed maps, but it does not optimize them. ElasticFusion is a 3D reconstruction based on a static environment, and it has the worst reconstruction results in most sequences. The error of filtering out dynamic factors and then inputting the sequences into BundleFusion for reconstruction is smaller than that of ElasticFusion. The proposed method adds dynamic processing based on the BundleFusion and optimizes the map, and the overall reconstruction error is small.

**Figure 9:**
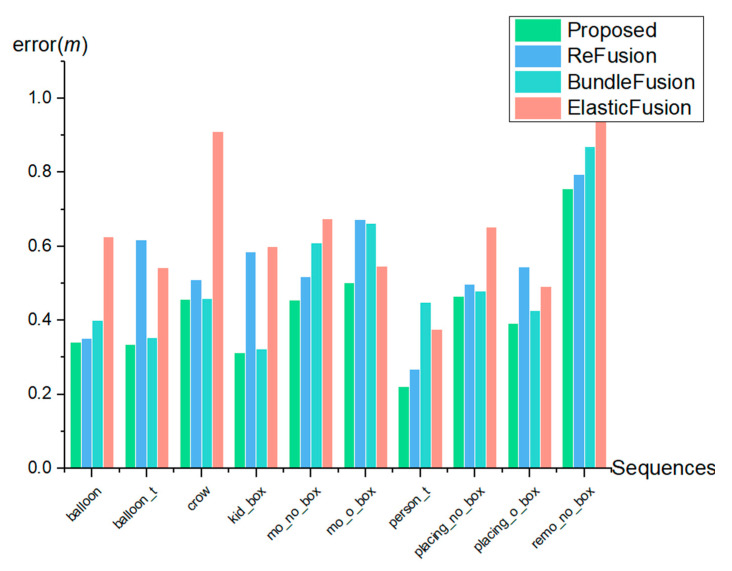
Surface reconstruction error (m).

**Figure 10:**
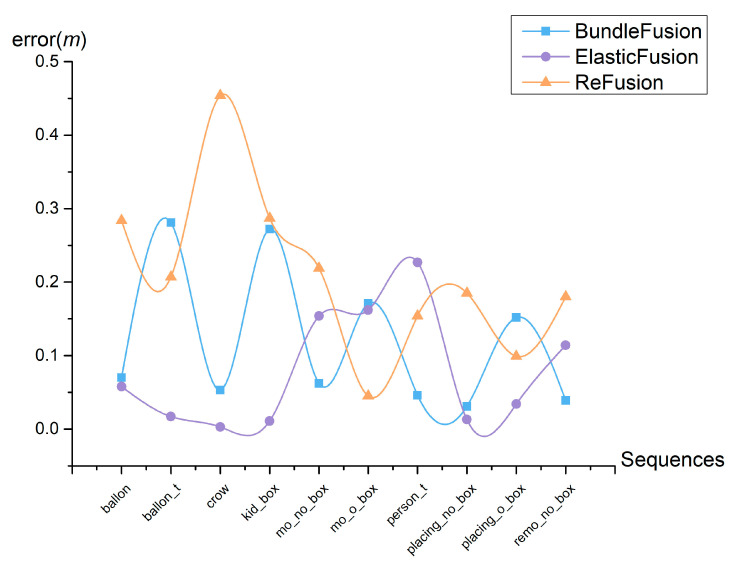
Errors between the proposed method and ReFusion, BundleFusion, and ElasticFusion.

To visualize the effect of the proposed method, the data from Table 1 are plotted in Figure 8, which shows that the reconstruction error between the 3D map by the proposed method and the real model is minimal. In Figure 9 and Figure 10, we shorten balloon_tracking with balloon_t, kidnapping_box with kid_box, moving_nonobstructing_box with mo_no_box, moving_obstructing_box with mo_o_box, person_tracking with person_t, placing_nonobstructing_box with placing_o_box, placing_obstructing_box with placing_o_box, and removing_nonobstructing_box with remo_no_box.

As can be seen in Figure 10, the reconstruction accuracy of the proposed method is higher than the reconstruction accuracy of the images after filtering out the dynamic factors and inputting them into ReFusion, BundleFusion, and ElasticFusion, respectively, for 3D reconstruction. This proves the effectiveness of the proposed method.

## 4. Discussion

To improve the accuracy of 3D maps in dynamic environments, a method for indoor dynamic environments is proposed in this manuscript. The TUM and BONN offline datasets were used in the experimental validation. In the framework of the BundleFusion-based algorithm, random fern coding was introduced to select key frames and remove dynamic factors more effectively. The experiments on the freiburg2_desk_with_person_validation sequence of the TUM dataset show that the 3D maps reconstructed based on this work have the least residual dynamic factors compared to the other three experimental methods compared. Experiments on the BONN dataset show that our method can significantly reduce the reconstruction error of the visual SLAM in an indoor dynamic environment.

However, the proposed method still has some shortcomings. For example, the texture features of the reconstructed 3D map are weaker than those of the real ground model.

We intend to apply the proposed method to indoor mobile robots. Fortunately, this does not affect the robot’s obstacle avoidance and navigation functions in the real environment. To provide the robot with a more perfect map for more work situations, the texture features of the reconstructed image will be further improved in subsequent work.

## 5. Conclusions

This study proposes a method based on BundleFusion to effectively filter out dynamic elements of indoor scenes and reconstruct static ones. The geometric residual method based on the TSDF model can effectively detect the dynamic factors and filter them out. However, it is not guaranteed that the filtered dynamic factors and the restored background images are identical to the static scene images, and the dynamic elements may be incompletely filtered. Therefore, a randomized fern is introduced to select keyframes, which reduces the influence of the residual dynamic factors on the visual SLAM system in the loop closure detection and relocalization, and improves the map reconstruction accuracy. In our future work, we aim to apply the proposed method to practical indoor mobile robot systems in order to solve real-world problems. At present, our team is cooperating with China Southern Power Grid Company Limited and intends to assemble an intelligent inspection robot. The proposed approach in this paper will be applied to the intelligent inspection robot.

## Figures and Tables

**Table 1:** Surface reconstruction error (m) evaluated on the BONN dataset by [18].

Sequences	Proposed	ReFusion	BundleFusion	ElasticFusion
balloon	0.342	0.351	0.400	0.626
balloon_tracking	0.336	0.617	0.353	0.543
crowd	0.457	0.510	0.460	0.911
kidnapping_box	0.313	0.585	0.324	0.600
moving_nonobstructing_box	0.455	0.517	0.609	0.674
moving_obstructing_box	0.501	0.672	0.663	0.546
person_tracking	0.222	0.268	0.449	0.376
placing_nonobstructing_box	0.466	0.497	0.479	0.651
placing_obstructing_box	0.392	0.544	0.426	0.491
removing_nonobstructing_box	0.756	0.795	0.870	0.936

## Data Availability

Not applicable.
